# Spinal Anesthesia With Short-Term Prophylaxis in a Patient With Hereditary Angioedema: A Case Report

**DOI:** 10.7759/cureus.93677

**Published:** 2025-10-01

**Authors:** Kazumasa Nishide, Takahiro Ishii, Yosuke Fujii, Tetsutaro Shinomura

**Affiliations:** 1 Department of Anesthesia, Otsu Red Cross Hospital, Otsu, JPN; 2 Department of Intensive Care, Otsu Red Cross Hospital, Otsu, JPN

**Keywords:** hereditary angioedema, laryngeal edema, plasma-derived c1 inhibitor, short-term prophylaxis, spinal anesthesia

## Abstract

Hereditary angioedema (HAE) is a rare genetic disorder characterized by recurrent episodes of edema affecting various body regions. Upper airway edema is a life-threatening condition that can progress to complete airway obstruction. Patients are particularly likely to encounter several triggering factors for HAE attacks during the preoperative period, necessitating specialized management.

An 80-year-old woman with an established diagnosis of HAE was scheduled to undergo bipolar hip arthroplasty following a femoral neck fracture. During pre-surgical hospitalization, she developed pharyngeal discomfort and hoarseness, which were diagnosed as HAE attacks. Immediate treatment with an intravenous plasma-derived C1 inhibitor (pdC1-INH) resulted in rapid resolution of the symptoms. Surgery was performed as scheduled under spinal anesthesia with the administration of a pdC1-INH as short-term prophylaxis. No postoperative HAE attacks were observed.

Surgical intervention in patients with HAE requires specialized care, and short-term prophylaxis may be useful in preventing HAE attacks.

## Introduction

Hereditary angioedema (HAE) is a rare, autosomal dominant inherited disorder characterized by recurrent attacks of angioedema. The C1 inhibitor not only suppresses the complement component 1 but also regulates multiple points in the plasma contact system, which is involved in coagulation and inflammation. A deficiency or dysfunction of the C1 inhibitor leads to an imbalanced activation of these systems, causing increased vascular permeability and resulting in edema. The prevalence of HAE varies by region but is estimated to be one in 50,000 worldwide [1]. Typical symptoms include skin swellings distinct from urticaria and abdominal pain caused by intestinal edema. Upper airway edema is a less frequent but life-threatening manifestation that can progress to airway obstruction. The perioperative period is a time of heightened risk for HAE attacks due to various triggering factors, requiring special management.

Herein, we report a patient with HAE who underwent bipolar hip arthroplasty under spinal anesthesia and remained free from postoperative HAE attacks after short-term prophylaxis. Written consent was obtained from the patient’s daughter for the publication of this case report.

## Case presentation

An 80-year-old woman presented to the emergency department with left leg pain and fever following a fall. Her medical history included chronic obstructive pulmonary disease, coronary artery disease, pulmonary hypertension secondary to chronic thromboembolism, and dementia. She had a history of cholecystectomy, appendectomy, left knee arthroplasty, right rotator cuff repair, cataract surgery, and tympanoplasty without perioperative complications. Medications included tiapride, azosemide, aspirin, atorvastatin, apixaban, danazol, and tranexamic acid.

Twenty-four years prior, the patient exhibited a C1 inhibitor activity of less than 25% and was diagnosed with type 1 HAE. The patient had been hospitalized repeatedly for abdominal pain and ileus; however, after initiating tranexamic acid and danazol, the frequency of abdominal symptoms decreased. Owing to dementia, recent information regarding symptoms and frequency could not be collected; however, it was unlikely that the patient had experienced serious HAE attacks that would have led to hospitalization.

**Figure 1:**
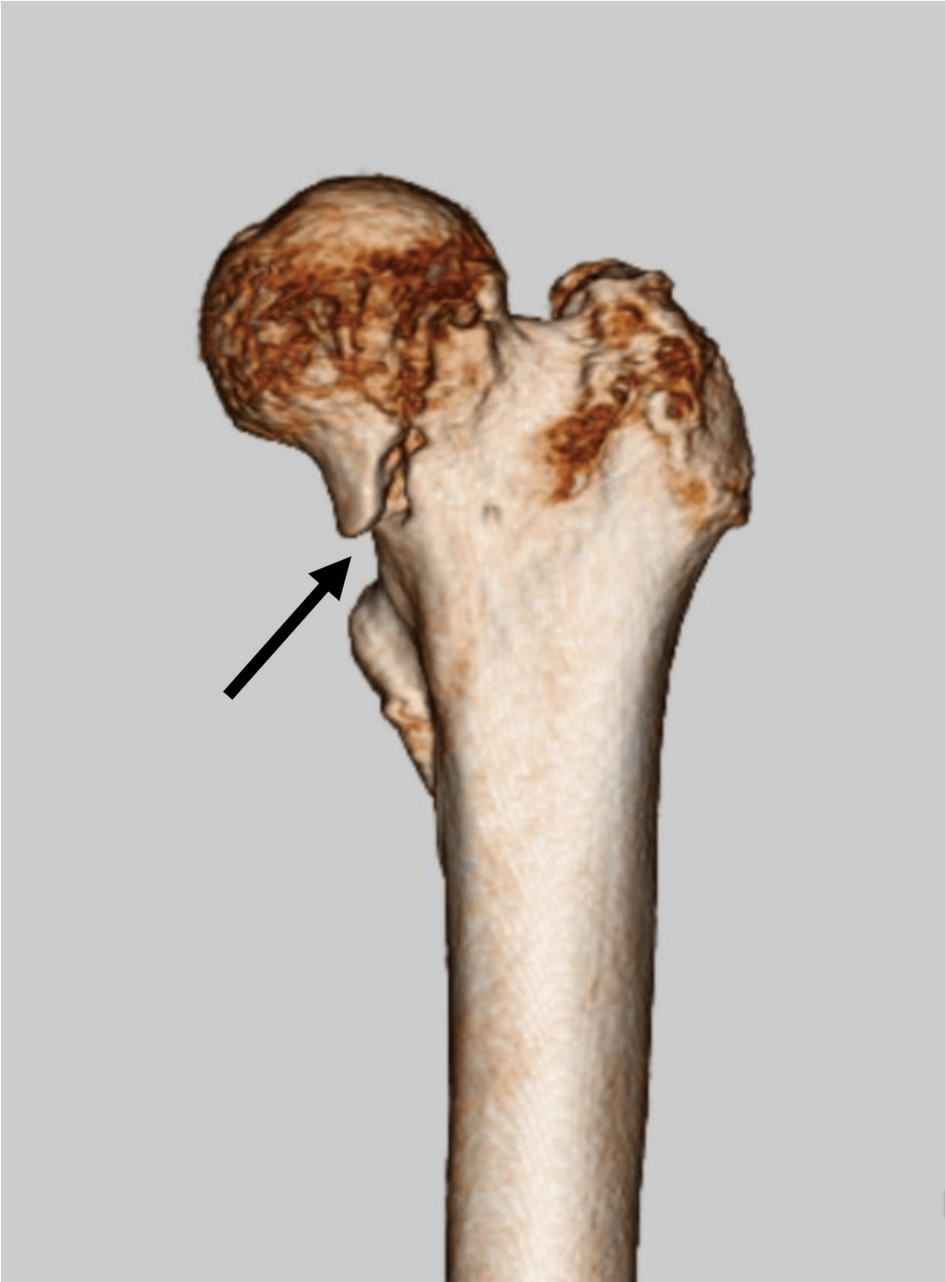
Preoperative three-dimensional computed tomography of left femur

After falling twice in her nursing home, the patient complained to her physician of fever and left leg pain. Radiography did not reveal any obvious bone fractures; however, her symptoms worsened, and she was referred to our emergency department. As shown in Figure 1, computed tomography revealed a fracture of the left femoral neck, and bipolar hip arthroplasty was performed.

On the third day of hospitalization, the patient complained of hoarseness upon waking. Thirty minutes later, pharyngeal discomfort and decreased oxygen saturation (SpO_2_ 88%) were observed. She was diagnosed with an HAE attack and treated with 1000 units of plasma-derived C1 inhibitor (pdC1-INH), and her symptoms soon abated. Subsequently, no HAE attacks recurred, and bipolar hip arthroplasty was performed on the eighth day.

Prior to surgery, 1000 units of pdC1-INH were administered as short-term prophylaxis. Surgery was performed under spinal anesthesia combined with sedation. Phenylephrine was administered as needed to maintain blood pressure. The surgery was completed without complications, and the patient was transferred to the intensive care unit (ICU). She experienced no HAE attacks in the ICU and was transferred to the general ward on the second postoperative day. The patient progressed uneventfully and was transferred to a rehabilitation hospital on the 35th day of hospitalization.

## Discussion

HAE is broadly classified into three categories: type 1, characterized by low C1 inhibitor levels; type 2, characterized by low functional C1 inhibitor levels; and HAE with normal C1 inhibitor levels [1]. It has been reported that the threshold level of C1 inhibitor required to prevent HAE attacks is 40% [2]. At initial presentation, the patient's C1 inhibitor level was <25%, which was sufficiently low to diagnose type 1 HAE. Follow-up laboratory records indicated that C1 inhibitor levels were maintained at 50%-60% for at least 10 years after the initiation of medication, consistent with a decrease in HAE attack frequency.

Symptoms of edema occur in various parts of the body, including swelling of the extremities, face, genitalia, and trunk, and abdominal pain caused by intestinal edema. Laryngeal edema is a rare manifestation that can lead to death due to upper airway obstruction. Although rare, accounting for only 0.9% of all HAE attacks, approximately 50% of patients with HAE experience laryngeal attacks during their lifetime [3,4].

**Figure 2:**
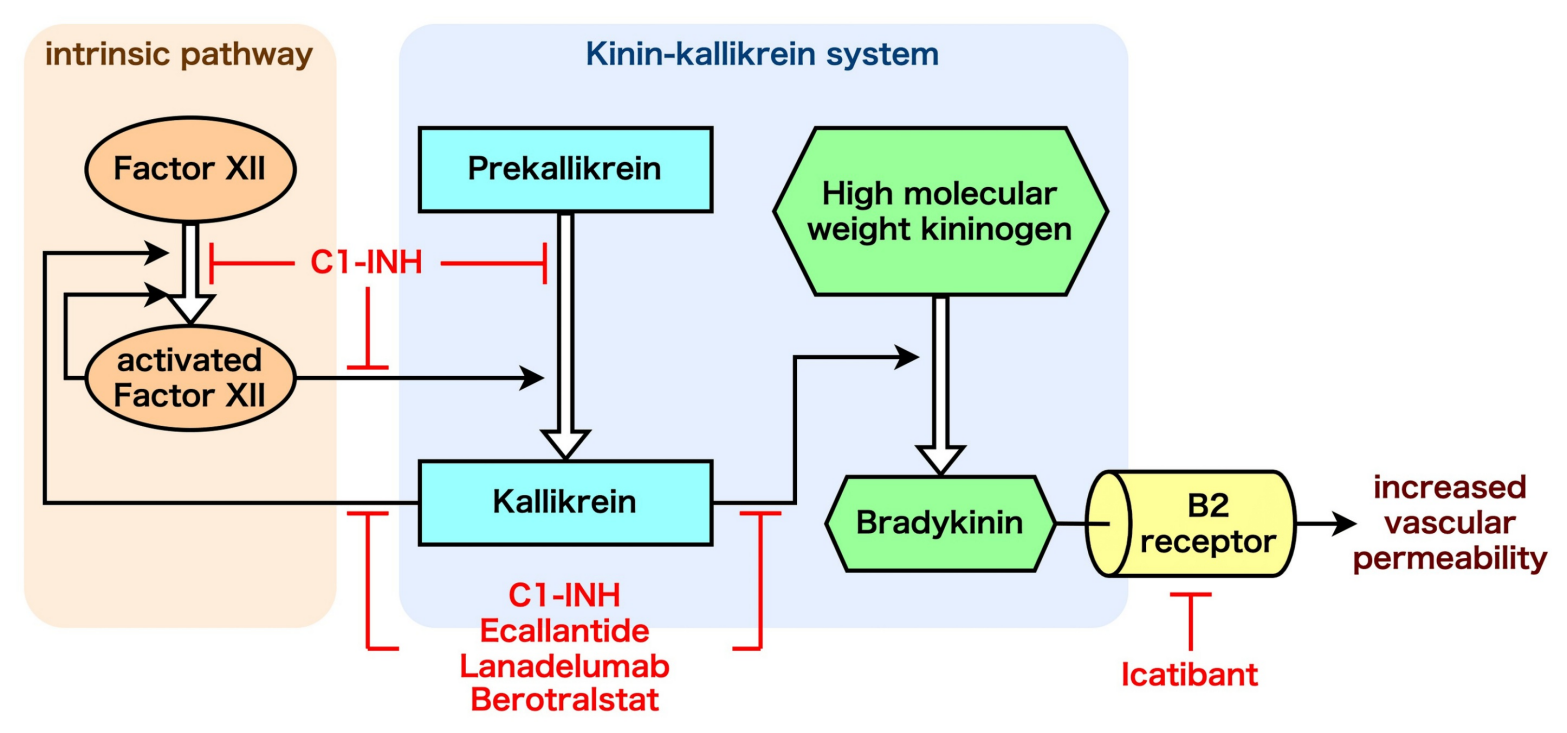
Mechanisms of hereditary angioedema (HAE) and related medications. This figure shows a simplified diagram of the contact activation system and the sites of action for several therapies for HAE. Activated factor XII (Factor XIIa) promotes the conversion of prekallikrein to kallikrein, which in turn cleaves high-molecular-weight kininogen (HMWK) to release bradykinin [3]. C1 inhibitor (C1-INH) negatively regulates this cascade. In HAE patients with deficient C1-INH activity, uncontrolled bradykinin production leads to increased vascular permeability via the B2 receptor. Several drugs target this pathway: ecallantide, lanadelumab, and berotralstat inhibit kallikrein, while icatibant blocks the B2 receptor. Image credit: Dr. Kazumasa Nishide.

As shown in Figure 2, C1 inhibitor dysfunction facilitates the activation of the intrinsic plasma contact system and the production of kallikrein, which, in turn, causes bradykinin production and local extravascular fluid transfer, particularly via the B2 receptor. As this pathway is not mediated by histamine or mast cells, common treatments such as adrenaline, corticosteroids, and antihistamines are ineffective. Medications that can be used to treat HAE attacks include pdC1-INH; recombinant human C1 inhibitor; ecallantide, a kallikrein inhibitor; and icatibant, a bradykinin B2 receptor inhibitor [1]. The first-line long-term prophylaxis includes intravenous or subcutaneous supplementation with C1 inhibitors; subcutaneous injection of lanadelumab, a plasma kallikrein inhibitor monoclonal antibody; and oral administration of berotralstat, a plasma kallikrein inhibitor. As was the case with this patient, androgens and antifibrinolytics have traditionally been used as long-term prophylaxis but are no longer first-line treatment in the latest guidelines [1]. For those at high risk of edema, especially those with anticipated triggers such as medical procedures, it is recommended to administer 1000 units or 20 units/kg of C1 inhibitor intravenously as short-term prophylaxis [1].

Upon admission, anesthesiologists, orthopedic surgeons, and hematologists decided to administer a pdC1-INH as short-term prophylaxis, considering that the absence of prior events does not suggest that future events will not occur [5]. As the drug was not in stock at that time, we ordered pdC1-INH from a wholesaler, and it arrived the following day. Fortunately, the drug arrived at the time of the HAE attack on day three. Specialty medications for rare diseases, including pdC1-INH, tend to be expensive, and because of the rarity of these conditions, they often have high disposal rates, making it difficult for hospitals to maintain their inventories. Therefore, establishing a system for prompt delivery from wholesalers when needed, or an inter-hospital network for stock flexibility, would be beneficial.

The triggers of HAE attacks include physical exertion, mental stress, mechanical trauma, infection, weather changes, menstruation, food intake, dental procedures, fatigue, and medical procedures [6]. Dental surgery and general anesthesia with endotracheal intubation can cause laryngeal edema [7]. Although the relationship between the trigger factor and the site of the HAE attack is not fully understood [6], many laryngeal edema cases occur spontaneously without a recognizable trigger [7]. Avoiding general anesthesia with airway manipulation during surgery for HAE patients seems reasonable, given the potential for serious consequences. Therefore, we decided to administer spinal anesthesia instead of general anesthesia, and adequate sedation was provided because mental stress can cause HAE attacks.

Limited data are available on the onset of HAE attacks owing to specific triggers. In particular, for facial and laryngeal edema after tooth extraction, Bork et al. reported the 6, 10, and 24 h as quartiles in a retrospective study [8]. As angioedema associated with medical procedures usually occurs within 48 h [1], the patient was followed up in the ICU for two nights after surgery.

**Figure 3:**
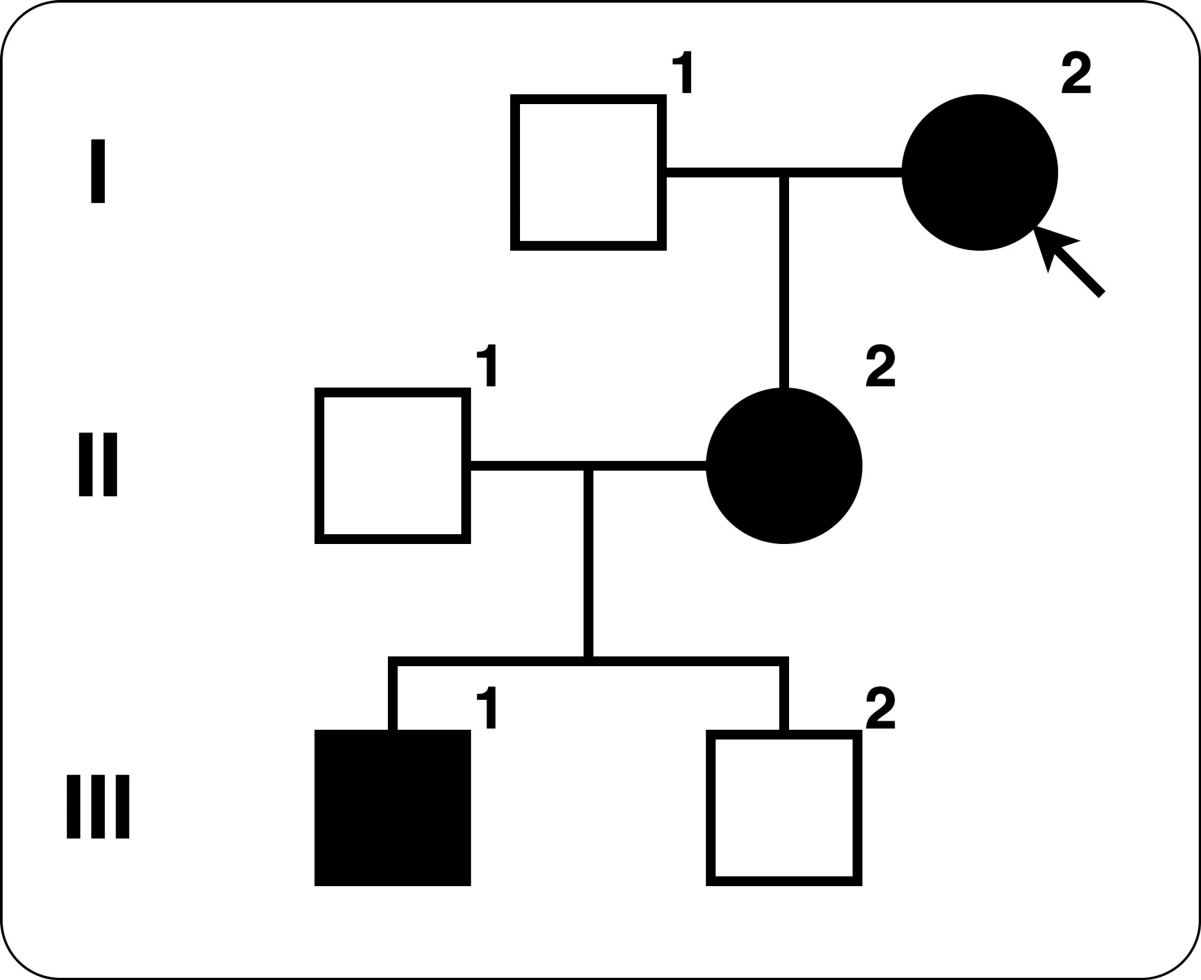
Pedigree chart of the patient. I-2 is the patient. III-2 is the patient's grandson who had undergone an emergency laparotomy at the same hospital. III-1 is another grandson who refused to confirm the diagnosis of HAE and experienced his first HAE attack with laryngeal edema. Image credit: Dr. Kazumasa Nishide.

As shown in Figure 3, the patient’s family includes several patients with HAE. Her grandson (III-2) had previously undergone emergency laparotomy at the same hospital. He was not diagnosed with HAE; however, his mother (II-2) and grandmother (I-2) had been diagnosed with HAE and administered pdC1-INH before surgery. He was eventually found not to have HAE, details of which were reported by Yazawa [9]. One of the siblings (III-1) refused to confirm the diagnosis of HAE. The sibling experienced his first HAE attack, including laryngeal edema, in his twenties and was rushed to the hospital, where his life was in danger. These episodes suggest that the families of patients with HAE are strongly encouraged to undergo testing because they could lose their lives due to laryngeal edema at any time, and a preliminary diagnosis and appropriate treatment could improve the situation.

## Conclusions

We report the case of a patient with HAE who underwent surgery under spinal anesthesia after short-term prophylaxis. Although progress has been made in identifying genetic abnormalities and molecular mechanisms and in developing new drugs for HAE, completely preventing HAE attacks is still not possible, and appropriate short-term prophylaxis is necessary in high-risk situations, including surgery.
